# Engineering a 3D-Bioprinted Model of Human Heart Valve Disease Using Nanoindentation-Based Biomechanics

**DOI:** 10.3390/nano8050296

**Published:** 2018-05-03

**Authors:** Dewy C. van der Valk, Casper F. T. van der Ven, Mark C. Blaser, Joshua M. Grolman, Pin-Jou Wu, Owen S. Fenton, Lang H. Lee, Mark W. Tibbitt, Jason L. Andresen, Jennifer R. Wen, Anna H. Ha, Fabrizio Buffolo, Alain van Mil, Carlijn V. C. Bouten, Simon C. Body, David J. Mooney, Joost P. G. Sluijter, Masanori Aikawa, Jesper Hjortnaes, Robert Langer, Elena Aikawa

**Affiliations:** 1 Center for Interdisciplinary Cardiovascular Sciences, Division of Cardiovascular Medicine, Department of Medicine, Brigham and Women’s Hospital, Harvard Medical School, Boston, MA 02115, USA; dewyvdvalk@gmail.com (D.C.v.d.V.); ponnie.wu@gmail.com (P.-J.W.); lhlee@bwh.harvard.edu (L.H.L.); jwen4@bwh.harvard.edu (J.R.W.); ahha@bwh.harvard.edu (A.H.H.); fbuffolo@bwh.harvard.edu (F.B.); maikawa@bwh.harvard.edu (M.A.); 2 Center of Excellence in Cardiovascular Biology, Division of Cardiovascular Medicine, Department of Medicine, Brigham and Woman’s Hospital, Harvard Medical School, Boston, MA 02115, USA; c.f.t.vanderven@umcutrecht.nl; 3 David H. Koch Institute for Integrative Cancer Research, Massachusetts Institute of Technology, Cambridge, MA 02142, USA; osfenton@mit.edu (O.S.F.); mtibbitt@ethz.ch (M.W.T.); jandrese@mit.edu (J.L.A.); rlanger@mit.edu (R.L.); 4 Wyss Institute for Biologically Inspired Engineering, Harvard University, Cambridge, MA 02138, USA; grolman@fas.harvard.edu (J.M.G.); mooneyd@seas.harvard.edu (D.J.M.); 5 John A. Paulson School of Engineering and Applied Sciences, Harvard University, Cambridge, MA 02138, USA; 6 Macromolecular Engineering Laboratory, Department of Mechanical and Process Engineering, ETH Zürich, 8092 Zürich, Switzerland; 7 Experimental Cardiology Laboratory, Department of Cardiology, University Medical Center Utrecht, Utrecht University, 3584 CX Utrecht, the Netherlands; a.vanmil@umcutrecht.nl (A.v.M.); j.sluijter@umcutrecht.nl (J.P.G.S.); jhjortna@umcutrecht.nl (J.H.); 8 Regenerative Medicine Center Utrecht, University Medical Center Utrecht, 3584 CT Utrecht, the Netherlands; 9 Department of Biomedical Engineering and Institute for Complex Molecular Systems, Eindhoven University of Technology, 5600 MB Eindhoven, the Netherlands; c.v.c.bouten@tue.nl; 10 Center for Perioperative Genomics, Department of Anesthesiology, Perioperative, and Pain Medicine, Brigham and Women’s Hospital, Harvard Medical School, Boston, MA 02115, USA; sbody@bwh.harvard.edu; 11 Channing Division of Network Medicine, Department of Medicine, Brigham and Women’s Hospital, Harvard Medical School, Boston, MA 02115, USA; 12 Department of Chemical Engineering, Massachusetts Institute of Technology, Cambridge, MA 02142, USA

**Keywords:** aortic valve, calcific aortic valve disease, calcification, mechanobiology, bioprinting, 3D printing, microdissection, nanoindentation

## Abstract

In calcific aortic valve disease (CAVD), microcalcifications originating from nanoscale calcifying vesicles disrupt the aortic valve (AV) leaflets, which consist of three (biomechanically) distinct layers: the fibrosa, spongiosa, and ventricularis. CAVD has no pharmacotherapy and lacks in vitro models as a result of complex valvular biomechanical features surrounding resident mechanosensitive valvular interstitial cells (VICs). We measured layer-specific mechanical properties of the human AV and engineered a three-dimensional (3D)-bioprinted CAVD model that recapitulates leaflet layer biomechanics for the first time. Human AV leaflet layers were separated by microdissection, and nanoindentation determined layer-specific Young’s moduli. Methacrylated gelatin (GelMA)/methacrylated hyaluronic acid (HAMA) hydrogels were tuned to duplicate layer-specific mechanical characteristics, followed by 3D-printing with encapsulated human VICs. Hydrogels were exposed to osteogenic media (OM) to induce microcalcification, and VIC pathogenesis was assessed by near infrared or immunofluorescence microscopy. Median Young’s moduli of the AV layers were 37.1, 15.4, and 26.9 kPa (fibrosa/spongiosa/ventricularis, respectively). The fibrosa and spongiosa Young’s moduli matched the 3D 5% GelMa/1% HAMA UV-crosslinked hydrogels. OM stimulation of VIC-laden bioprinted hydrogels induced microcalcification without apoptosis. We report the first layer-specific measurements of human AV moduli and a novel 3D-bioprinted CAVD model that potentiates microcalcification by mimicking the native AV mechanical environment. This work sheds light on valvular mechanobiology and could facilitate high-throughput drug-screening in CAVD.

## 1. Introduction

Calcific aortic valve disease (CAVD) is the most prevalent heart disease, affecting more than ¼ of individuals over age 65 in the Western world [1]. Despite this high prevalence and many risk factors shared with atherosclerosis, there are no pharmacological therapies for CAVD—aortic valve (AV) replacement currently is the sole treatment option. The healthy AV is composed of three semilunar leaflets. The leaflets are comprised of three stacked layers, each with its own unique extracellular matrix (ECM) composition: the collagen-rich fibrosa layer, the proteoglycan-rich spongiosa, and the elastin-rich ventricularis [2]. As CAVD progresses, the leaflets of the AV become fibrotic and calcify as their constituent cell population of valve interstitial cells (VICs) undergo myofibrogenic and osteogenic differentiation [2]. Calcification is likely to initiate via aggregation of nanoscale calcifying vesicles into larger microcalcifications [3,4], which eventually form extensive regions of ectopic calcific nodules that impair AV opening/closure, leading to heart failure and death [5]. Importantly, the onset and progression of CAVD is layer-specific: it preferentially initiates within the fibrosa and progresses to the spongiosa, while the ventricularis is largely unaffected until the final stages of disease [6,7].

Preferential disease development in the fibrosa may be a result of VICs sensing and responding to the biomechanics of their microenvironment. Similar to mesenchymal stem cells [8], pathological differentiation of normally quiescent VICs to myofibroblasts and osteoblasts is regulated in vitro by the local mechanics (e.g., compressive stiffness) of the ECM [9,10,11]. Tensile mechanical properties of whole human AV leaflets have been studied [12] as well as those of microdissected human fibrosa and ventricularis [13,14]. Micropipette aspiration has been used to measure the Young’s modulus of normal porcine fibrosa and ventricularis [10]; however, the only measurements of spongiosa biomechanics originate from atomic force microscopy of layers on thin porcine leaflet cryosections [15]. Importantly, all three layers of the human AV have not been separated and subjected to measurements of local compressive stiffness. VIC mechanosensitivity is likely a key limiting factor in current in vitro platforms for high-throughput drug discovery, which are based on tissue culture polystyrene (TCPS) with high, non-physiological stiffness. Existing in vitro models of CAVD cannot account for mechanosensitive cellular responses that may modulate otherwise-drugable signaling axes in vivo.

Although many putative therapeutic targets have been identified in recent years [16,17], the mechanosensitive nature of VICs and layer-specific biomechanics of the native AV necessitate a suitably scalable in vitro system that recapitulates key aspects of the valvular architecture and microenvironment (e.g., layer-specific mechanical properties) to reliably screen and test novel targets. To this end, a variety of soft gel substrates have been utilized to examine VIC phenotype and function, including those based on poly(ethylene-glycol) (PEG) [18], PEG-dimethacrylate-poly(l-lactide) (PEGdma-PLA) [15], polyacrylamide [10], and collagen [9]. We and others have shown previously that hybrid GelMA/HAMA hydrogels are able to maintain VICs in a quiescent fibroblastic state [19], while also supporting differentiation of VICs towards diseased phenotypes after exogenous delivery of pathological cytokines or other calcifying stimuli [11,20]. 3D-bioprinting has been shown to provide superior cell seeding and cell attachment compared with traditional scaffold biofabrication techniques, along with lower gel-to-gel variability and hands-off (high-throughput) fabrication [reviewed in S.V. Murphy et al. and C.F. van der Ven et al. [21,22]]. 3D-bioprinting has been utilized to fabricate complex tissues in vitro (e.g., co-culture tumor models, branched vascular trees, and cartilaginous structures such as ears and trachea [23,24,25,26]), and is compatible with GelMA-based hydrogels [27]. This technique has been utilized to manufacture large-format valvular conduits [28] and flow phantoms [29] but has not been applied to model the complexities of the valvular microenvironment.

In the present study, we first performed systematic microdissection and mechanical testing of human AV tissue, then coupled this data with 3D-bioprinting and mechanical characterization of a tunable hydrogel system to mimic the native valve. Here, we demonstrate (1) the first approach to separate all three layers of the human AV; (2) nanoindentation measurements of layer-specific biomechanical properties of the native human AV; (3) direct recapitulation of layer-specific Young’s moduli using a 3D GelMA/HAMA hydrogel system; (4) bioprinting of 3D hydrogels with encapsulated primary human VICs; (5) controllable formation of layer-specific microcalcification and ECM degradation by VICs; and (6) bioprinting of multi-layered 3D hydrogel AV constructs. Together, these data establish a novel 3D model for studying the mechanisms of valvular diseases and a platform suitable for high-throughput screening in CAVD.

## 2. Materials and Methods

### 2.1. Layer Separation of CAVD Leaflets is Confirmed by Histological Evaluation

AV leaflets were obtained from patients undergoing surgical valve replacement at Brigham and Women’s Hospital (Boston, MA, USA) as a result of AV calcification and/or stenosis. Leaflets were obtained and utilized in accordance with protocols approved by the Institutional Review Board (IRB protocol #2011P001703/PHS). After removal, leaflets were kept on ice in Dulbecco’s Modified Eagle’s Medium (DMEM, Lonza, Walkersville, MD, USA) for a maximum of 1 h, then stored for a maximum of 10 h in DMEM at 37 °C/5% CO_2_. Leaflet layers were microdissected as described previously [13,14] with modifications to additionally obtain spongiosa samples. Non-calcified regions of the CAVD leaflets were selected by gross morphology and absence of any palpable regions of stiff/brittle calcific deposits, and cut using #10 scalpels (Fine Science Tools, North Vancouver, BC, Canada). In a recent paper from our laboratory [17], we demonstrate the high specificity of this stage-separation approach in human AVs by proteomics (LC-MS/MS) and transcriptomics. Regions that were to undergo testing on whole (intact) leaflets were marked with tissue-marking dye (TMD, General Data Company Inc., Cincinnati, OH, USA) on the fibrosa side to ensure orientation. Regions selected for layer separation were pinned through the ventricularis layer to a cork dissecting board using 30 g needles, with the fibrosa surface facing upward. Samples were submerged in DMEM during the procedure. Fibrous interconnections within the spongiosa that ran between the ventricularis and fibrosa were exposed by gently lifting the fibrosa with forceps. Under a dissecting microscope, these fibrous interconnections were cut by microscissors at the bottom of the fibrosa, and fibrosa segments were marked by TMD on the aortic-facing surface to ensure testing of only the collagenous fibrosa layer. Segments were gently transferred to a separate container with DMEM and kept on ice. The exposed spongiosa layer was gently grasped, cut away from the ventricularis layer, and transferred to DMEM on ice. The remaining ventricularis layer was marked on the ventricle-facing side with TMD and placed in DMEM on ice. Samples underwent mechanical testing within <10 h of layer separation. Following mechanical testing, samples were embedded in Optimal Cutting Temperature compound (OCT, Tissue-Tek Sakura, Torrance, CA, USA), cryopreserved on a dry ice and isopentane bath, then sectioned and stained with Movat’s pentachrome (MP, American MasterTech—KTRMP) to confirm sample layer specificity and tissue viability.

Cryoslides were dried and sections were fixed in formalin and rinsed with water. Using the MP kit, the sections were immersed in Verhoeff’s elastic stain for 15 min and differentiated with 2% ferric chloride. The slides were then placed in Alician blue solution for 10 min, Crocein scarlet-acid fuchsin for 2 min, and alcoholic saffron solution for 15 min, clearing and rinsing the slides between stains. The slides were finally cleared in xylene and coverslipped using a xylene-based mounting media. Three non-serial 10 μm-thick sections per sample were stained, and imaged with an Eclipse 50i microscope (Nikon Instruments Inc., Melville, NY, USA). Spongiosa samples that contained collagen-rich areas or elastin-rich areas larger than 5% of the total sample area or fibrosa samples with >5% proteoglycan content were excluded from final analyses as contaminants from the adjacent layers.

### 2.2. Methacrylated Gelatin (GelMA) and Methacrylated Hyaluronic Acid (HAMA) Synthesis and Characterization

GelMA and HAMA were prepared as described previously [30,31]. In brief, gelatin (20.0 g) from porcine skin (Sigma-Aldrich, St. Louis, MO, USA, G2500) was suspended in deionized water (200 mL) in a 500-mL round bottom flask with moderate stirring for 1 h. The mixture was heated to 50 °C and was stirred until the gelatin was completely dissolved. Methacrylic anhydride (12.0 g) (Sigma-Aldrich, 276685) was then added to the flask, and the mixture was stirred at 50 °C for 1.5 h. The product was transferred to 50 mL conical tubes and centrifuged at 3500× *g* for 5 min. The supernatant was decanted into a beaker, leaving behind an opaque solid at the bottom of the conical tubes. The supernatant was then diluted with two volumes of 40 °C deionized water and was transferred to dialysis tubing (10 kDa MWCO, SpectraPor 7, Spectrum Laboratories, Rancho Dominguez, CA, USA, 123120). This tubing (containing the GelMA supernatant) was dialyzed against 3500 mL of deionized water at 40 °C for seven days, with the water changed twice per day. The contents of the dialysis tubing were then transferred to a beaker and pH adjusted to 7.4 using a 1 M solution of NaHCO_3_. The solution was sterile filtered with a 0.2 μm vacuum filtration unit and a polyethersulfone (PES) membrane, transferred to 50 mL conical tubes, snap frozen on liquid nitrogen, and lyophilized until complete dryness (approximately 10–14 days) to produce GelMA as a white solid powder.

For HAMA synthesis, sodium hyaluronate (1.0 g) (Lifecore Biomedical, Chaska, MN, USA, HA40K) was dissolved in 1X phosphate-buffered saline (PBS) (100 mL) in a 250 mL round bottom flask and cooled to 4 °C. Methacrylic anhydride (1.0 mL) was added and stirred at 4 °C for 24 h; pH was maintained between 8.0 and 10.0 at the beginning, middle, and end of the reaction period using aliquots of 5 M NaOH. After 24 h the reaction was transferred to 50 mL conical tubes, centrifuged at 3500× *g* for 5 min, and the supernatant was decanted into dialysis tubing (10 kDa MWCO, SpectraPor 7). The tubing containing the HAMA solution was dialyzed against 3500 mL of deionized water at 4 °C for seven days, with the water changed twice per day. Tubing contents were transferred to a beaker and the pH adjusted to 7.4 using a 1 M solution of NaHCO_3_. The solution was sterile filtered with a 0.2 μm vacuum filtration unit with a PES membrane, transferred to 50 mL conical tubes, snap frozen on liquid nitrogen, and lyophilized until complete dryness (approximately 10–14 days) to produce HAMA as a white solid powder.

Pre-polymer solutions were mixed prior to printing. Measured weights of lyophilized GelMA, lyophilized HAMA, and lithium phenyl-2,4,6-trimethylbenzoylphosphinate (LAP, Tokyo Chemical Industry Co., Portland, OR, USA) were dissolved in PBS at 80 °C to form 20 wt %, 3 wt %, and 5 wt % solutions, respectively. GelMA solution sonicated at 37 °C, and the pH of HAMA was adjusted to 7.5 using 1 M HCl. Solutions were reheated to 80 °C for 20 min and sterile-filtered using a 0.2 μm syringe filter. Final solutions were stored at 4 °C and warmed to 37 °C prior to each experiment. For the fabrication of hybrid hydrogels, the 20 wt % GelMA, 3 wt % HAMA, and 5 wt % LAP solutions were mixed in PBS at 37 °C. This yielded hybrid hydrogel pre-polymer solutions with concentrations of 0.3% (*v*/*v*) LAP, 1% (*v*/*v*) HAMA, and 5% (*v*/*v*), 6.67% (*v*/*v*), 8.33% (*v*/*v*), or 10% (*v*/*v*) GelMA.

### 2.3. 3D-Bioprinting of Hybrid Hydrogels

#### 2.3.1. Single-Layer Hydrogel Constructs

Constructs were designed in Tinkercad (AutoDesk, Inc., San Rafael, CA, USA), and encoded using Repetier-Host (version 2.0.0; Hot-World GmbH & Co. KG, Willich, Germany), and Sublime Text 3 (Sublime HQ, Pty Ltd., Darlinghurst, NSW, Australia). 3D-bioprinting was performed using the Inkredible+ (Cellink, Cambridge, MA, USA). Pluronic gel (Pluronic F-127; Allevi, Philadelphia, PA, USA) was printed as a cylindrical mold (outer diameter = 9.0 mm, inner diameter = 8.6 mm, height = 1.5 mm) using a stainless steel needle nozzle (JG27-0.25HPX; Jensen Global Inc., Santa Barbara, CA, USA) at a fill density of 95%, layer height of 0.1 mm, printing speed of 4mm/s, and printing pressure of 320 kPa.

The second extruder was filled with the required hydrogel pre-polymer compositions and heated to 37 °C. A hydrogel disc was then printed inside the mold from the second extruder using a 23 g stainless steel nozzle (Fisnar 5901005, Ellsworth Adhesives, Germantoun, WI, USA) by opening the valve of the second extruder for 40 ms at 15–20 kPa. Crosslinking the pre-polymers for 30 or 90 s with 365 nm UV light produced 8.6 mm × 1.0 mm hydrogel discs. The UV light was calibrated to an intensity of 2.5 mW/cm^2^ using a radiometer (85009, Sper Scientific Direct, Scottsdale, AZ, USA). After printing, the Pluronic gel was dissolved by washing in 37 °C PBS. Hydrogel porosity was evaluated by embedding and cryosectioning as described above. Pore structure was assessed by staining with Natural Blue food dye (Whole Foods Market, Austin, TX, USA). Bright field microscopy images were then taken using an Eclipse 50i microscope (Nikon Instruments, Melville, NY, USA). Average pore size was then measured with ImageJ (National Institutes of Health, Bethesda, MD, USA, version 1.51s). To perform swelling ratio testing, acellular hydrogels were printed and crosslinked. The Pluronic mold was then cut away, and gels were weighed immediately then immersed in PBS for 24 h at room temperature, at which point they were quickly blotted dry of surface liquid and weighed again.

#### 2.3.2. Dual-Layer Hydrogel Constructs

The Pluronic mold was printed as described above. A hydrogel pre-polymer solution of 0.3% LAP, 5% GelMA, and 1% HAMA at 37 °C was loaded into the second extruder. The first layer was printed by opening the valve of the second extruder for 30 ms at 15–20 kPa. After crosslinking the first layer for 60 s at 2.5 mW/cm^2^, the extruder was raised 1 mm and a second layer was printed on top by opening the valve of the second extruder for 30 ms. After 5 min incubation at 37 °C, the combined layers were crosslinked together for an additional 30 s. To visualize the layer interface, 70.000 MW lysine-fixable Dextrans labeled with Texas Red or Fluorescein (Thermo Fischer Scientific, Waltham, MA, USA) were mixed into each hydrogel layer, and the constructs were imaged by confocal (Nikon) in cross-sectional z-stacks to assess layer-layer integration.

### 2.4. Mechanical Testing of CAVD Leaflets and Hydrogels

Mechanical testing was performed using an Agilent G200 nanoindenter (Agilent, Santa Clara, CA, USA) with a 90° diamond conical probe tip with a 50 μm radius (DCMII, Micro Star Technologies, Huntsville, TX, USA) to enable measurement of bulk properties [32]. The tip area function was calibrated using fused quartz, and a punch diameter of 45.153 μm was calculated at a 5 μm pre-compression depth. A 3 × 3 array of indents with 200 μm spacing was generated. Tests were run as dynamic indentations to afford the complex shear modulus under the shear mode [33] at room temperature at a constant frequency of 110 Hz. Storage modulus G′ was measured as the energy stored during one oscillation cycle, and loss modulus G′′ was measured as the energy dissipated during an oscillation cycle [34]. The loss tangent (tan δ) was measured as the ratio between G′′ and G′. The complex modulus G* was calculated using:

(1) $$\left|G^{*}\right|=\sqrt{G^{\prime }{}^{2}+G^{\prime \prime }{}^{2}}$$

Poisson’s ratio (v) was estimated for both the leaflet layers and the hydrogels using two different-sized conical probes and were found to be approximately 0.5, corresponding to values confirmed in the literature for bulk measurements of hydrogel polymers [35] and AV leaflet tissue [36]. Using the rubber elasticity theory, Young’s modulus (E) was calculated as:

(2) $$E=2G^{*}\left(1+v\right)$$

The median of nine indents was taken for every sample. For the mechanical measurement of dual-layer hydrogels, two tests were performed. First, hydrogels were tested from both sides to evaluate layer-to-layer differences. Second, the cross-section of the hydrogels was tested along the *z*-axis using 10 nanoindentation measurements equally spaced from the top of the fibrosa-like side to the bottom of the spongiosa-like side.

Unconfined compression testing was also performed on selected hydrogels using an Instron 5566 (Instron, Norwood, MA, USA). Compression occurred at a rate of 1 mm/minute between two parallel steel plates, and the Young’s modulus was calculated from the slope of the linear region of the loading curve.

### 2.5. Human Aortic Valvular Interstitial Cell (VIC) Isolation, Culture, and Encapsulation in Hydrogels

CAVD AV leaflets were obtained as described above, and non-diseased AV leaflets were obtained from patients undergoing heart transplantation surgery due to cardiomyopathy at UMC Utrecht (the Netherlands). Leaflets were obtained there in accordance with protocols approved by the Medical Ethical Assessment Committee of UMC Utrecht.

VICs from CAVD AV leaflets and non-diseased AV leaflets were isolated as previously described [9,37]. In brief, CAVD leaflets were incubated for 3 h in a 10 mL collagenase solution at 37 °C, 5% CO_2_, homogenized with a serological pipette and passed through a cell strainer (40 μm). The digested tissue was centrifuged, the supernatant was aspirated, and the pellet was resuspended in 5 mL of VIC cell culture media. The cells were centrifuged a second time, resuspended in 10 mL media, and plated in a T75 culture flask. Non-CAVD leaflets were incubated for 45–50 min at 37 °C in 8% collagenase solution in PBS supplemented with 1% FBS and 2% gentamycin. The digested tissue was passed through a cell strainer (100 μm), rinsed 3 × 5 mL in DMEM, and centrifuged. The supernatant was aspirated, and the pellet was resuspended in 5 mL VIC cell culture media and plated in 6-well plates. VIC culture media (DMEM, ThermoFisher, Grand Island, NY, USA) was supplemented with 10% (CAVD VICs) or 15% (non-diseased VICs) fetal bovine serum (Gibco), 5% human serum (first three passages of non-diseased VICs only), and 1% Penicillin/Streptomycin (P/S, Gibco). Media was refreshed every 48 h, DMEM was supplemented with 10% FBS and 1% P/S after passage three regardless of VIC type, and VICs between passage five and six were used for further experiments. VICs were incorporated in the hydrogel pre-polymers by mixing VICs and media (to a final in-gel concentration of 10 × 10^6^ cells/mL) with a 10 wt % GelMA, 3 wt % HAMA, and 5 wt % LAP solution at 37 °C to form a 5% GelMA, 1% HAMA, and 0.3% LAP hydrogel. As a 2D control, VICs were also cultured on tissue culture polystyrene (TCPS) in a 24-well plate. One day after printing, hydrogels were switched to normal media (5% FBS, 1% P/S) or osteogenic media (NM supplemented with 10 nM dexamethasone, 10 ng/mL ascorbic acid, and 10 mM β-glycerolphosphate) as previously described [4] for up to 14 days. Media was changed every 48 h for all 3D hydrogels and 2D controls.

### 2.6. Calcification and Apoptosis Assays

A near infrared fluorescence (NIRF) imaging agent (OsteoSense 680EX; PerkinElmer, Waltham, MA, USA) was used to visualize nano- and microcalcification. OsteoSense 680EX was added to the cell culture media at a 1:100 dilution and cells were incubated overnight at 37 °C, 5% CO_2_ for 12 h prior to imaging. Apoptosis was assessed by Click-iT TUNEL (Thermo Fisher Scientific, Waltham, MA, USA) assays according to the manufacturer’s instructions. Confocal imaging (Nikon A1) was performed as follows: three hydrogels per condition were imaged with three z-stacks (10 μm/slice) per hydrogel. Z-stacks were compressed into maximum intensity projections in ImageJ and positive stain area was quantified.

### 2.7. Analysis of VIC Remodeling Capability

Hydrogels were snap frozen in OCT as described above and 10 μm cryosections were made. Ability of VICs to remodel ECM was gauged by collagen production and its degradation by matrix metalloproteinase 9 (MMP-9). Sections were stained for collagen using the CNA35 probe by incubating sections (1:50 in PBS) for 1 h at 37 °C [38]. MMP-9 immunofluorescence was performed as follows: sections were permeabilized with 0.1% Triton-X, then dried and fixed in 4% paraformaldehyde for 5 min. Slides were rinsed in water and PBS, and endogenous peroxidase activity was blocked with 0.3% hydrogen peroxide. After rinsing, sections were blocked in 4% donkey and goat serum (D9663, Sigma, St. Louis, MO, USA, S-1000, Vector, Burlingame, CA, USA) and then incubated with an anti-MMP-9 mouse (NBP2-13173g, 1:100, Novus, Littleton, CO, USA) primary antibody for 90 min at room temperature, followed by an Alexa Fluor 488-conjugated (A11017, Invitrogen, Carlsbad, CA, USA) secondary antibody. Sections were washed in PBS and mounted in mounting media with DAPI (H-1500, Vector Laboratories, Burlingame, CA, USA) to stain nuclei. Confocal imaging was performed as above, and positively stained cells were quantified by manual counting.

### 2.8. Electron Microscopy

Hydrogels were fixed and stored in a buffer of 2% gluteraldehyde, 0.115 M sucrose, in 0.1 M sodium cacodylate (Electron Microscopy Science, Hatfield, PA, USA, 11653) until use. Constructs were dehydrated in a series of 30%, 50%, 70%, 90%, 100%, 100%, and 100% ethanol on ice for 15 min each, prior to critical point drying (Critical Point Dryer Tousimis 931 GL; Tousimis, Rockville, MD, USA). Constructs were then coated with platinum/palladium in a sputtering system (EMS 300 T D Dual Target Sequential Sputtering System; Electron Microscopy Systems, Hatfield, PA, USA). Scanning electron microscopy (SEM) images were taken on a field emission scanning electron microscope (Supra55VP; Zeiss, Thornwood, NY, USA).

### 2.9. Statistical Methods

Nanoindentation data are presented as the median value per tissue sample, with each data point representing nine nanoindentation measurements per sample. Other quantitative data are presented as mean ± standard error. Two-tailed, one-sample *t*-tests were used to compare G′′/G′ values vs. the theoretical value of 1, Student’s *t*-tests were performed for two-group comparisons, and one-way or two-way ANOVA with Tukey’s post-hoc HSD test were used as appropriate to evaluate statistically significant differences in multiple group comparisons (R or GraphPad Prism 7, GraphPad Software, La Jolla, CA, USA).

## 3. Results

### 3.1. Leaflet Layer Separation of Human Aortic Valves by Microdissection

**Figure 1:**
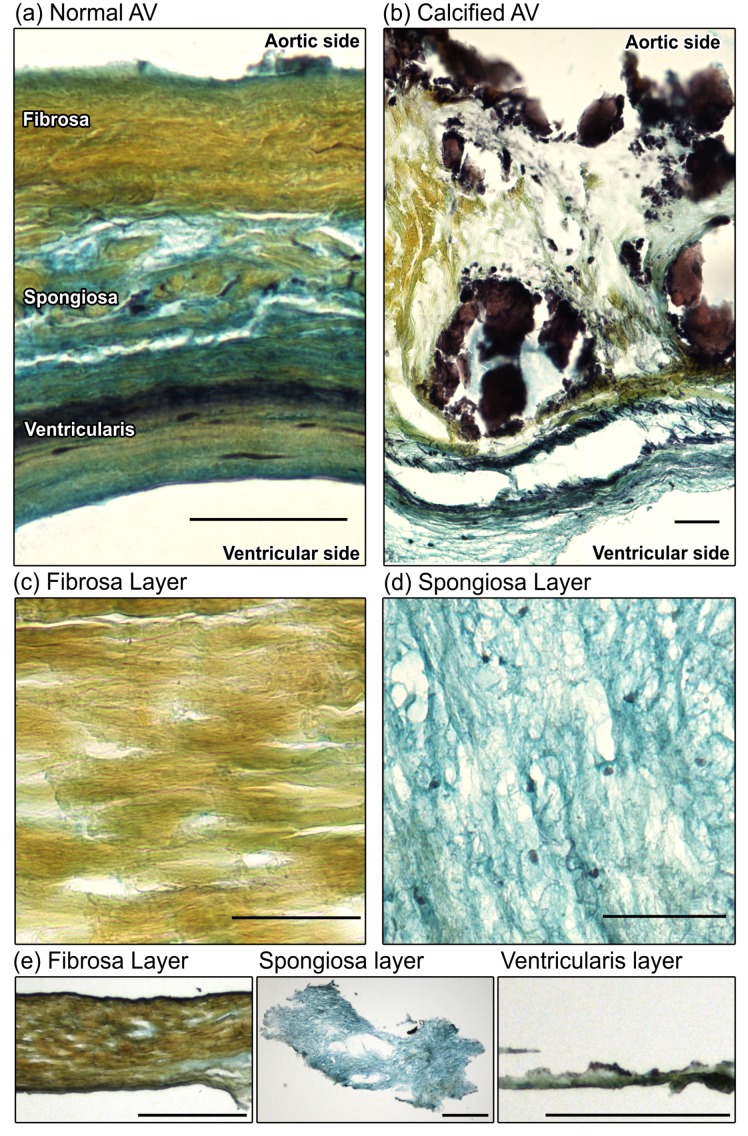
Movat’s pentachrome staining of structural organization in a circumferential cross-section of a human AV leaflet. Yellow = collagen, blue = glycosaminoglycans (GAGs), black = elastin, dark brown/black = calcification: (**a**) Tri-layered arrangement of the fibrosa (collagen-rich), spongiosa (GAG-rich), and ventricularis (elastin-rich) layers in a healthy leaflet; (**b**) Disruption of the leaflet layers by calcifications (black) and fibrosis (yellow regions) in CAVD; (**c**,**d**) Staining of collagen in the dissected fibrosa layer and GAGs in the dissected spongiosa layer confirmed distinct layer separation; scale bar = 50 μm; (**e**) Low-magnification images of microdissected fibrosa, spongiosa, and ventricularis layers; scale bar = 500 µm.

The human AV is composed of three distinct layers with unique ECM composition and VIC phenotypes [17]. The fibrosa layer is composed primarily of circumferentially oriented collagen fibers (yellow by Movat’s); the spongiosa layer sits in the middle of the leaflet and is rich in hydrated proteoglycans (blue-green); the ventricularis is rich in radially oriented elastin fibers [39] (black; Figure 1a). As CAVD progresses, calcification and fibrosis develop preferentially in the fibrosa layer [6,7] and the layers themselves are thickened and disrupted (Figure 1b). We separated non-calcified leaflet layers by microdissection and confirmed that dissected layers were successfully divided by Movat’s pentachrome staining, which identified collagen-rich samples from the fibrosa layer and glycosaminoglycan-rich samples dissected from the spongiosa layer (Figure 1c–e).

### 3.2. 3D-Bioprinting of Hybrid Hydrogel Constructs

**Figure 2:**
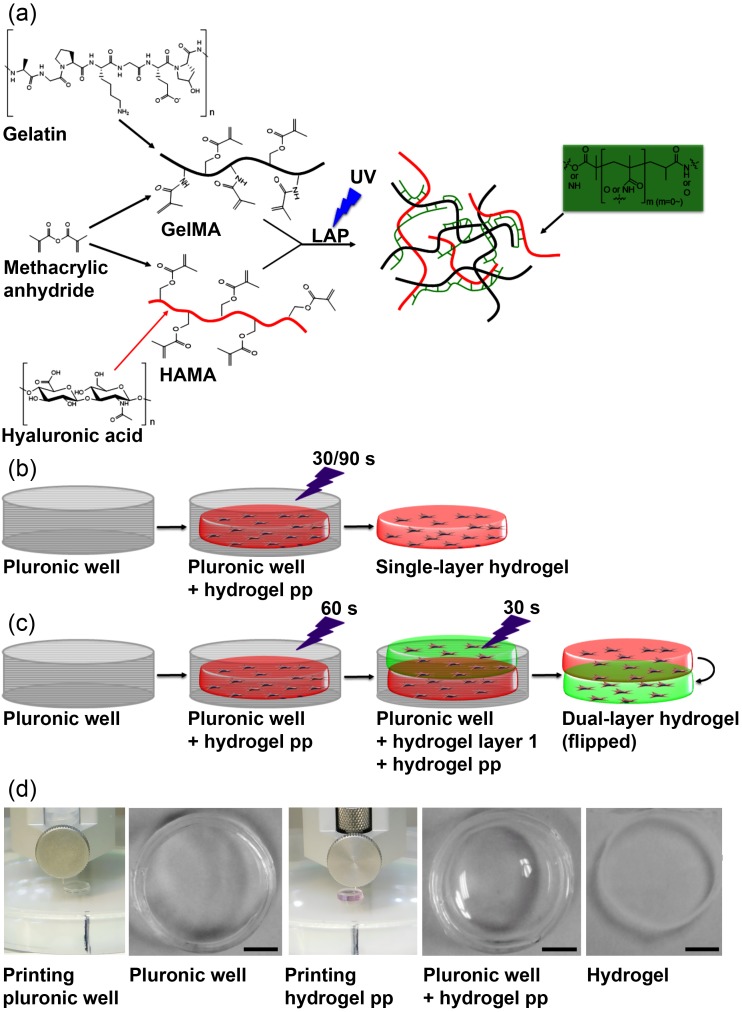
Bioprinting of hybrid hydrogel constructs using methacrylated gelatin (GelMA) and methacrylated hyaluronic acid (HAMA): (**a**) Schematic of hybrid GelMA/HAMA hydrogel synthesis and gelation. Black = gelatin, Red = hyaluronic acid, green = crosslinks, UV = photocrosslinking with 365 nm UV light at 2.5 mW/cm^2^; (**b**,**c**) 3D printing of single- and dual-layer hydrogel constructs by printing a Pluronic ring, followed by hydrogel pre-polymer (pp) inside, and photocrosslinking for a total of 30 and/or 90 s per layer; (**d**) Representative images of (left to right): 3D bioprinting the Pluronic ring, the completed Pluronic ring, 3D bioprinting the hydrogel pre-polymer into the Pluronic ring, the completed Pluronic ring + printed hydrogel pre-polymer, and hydrogel after crosslinking and washing away the Pluronic (right). Scale bar = 2 mm.

To produce an in vitro model of human CAVD that was capable of recapitulating AV leaflet layer-specific mechanical properties, we employed a tunable hydrogel system that we had previously shown to be capable of maintaining porcine VIC quiescence under basal conditions in 3D [19]. Our platform utilized methacrylated gelatin (GelMA), methacrylated hyaluronic acid (HAMA), and the UV photoinitiator lithium phenyl-2,4,6-trimethylbenzoylphosphinate (LAP) (schematic of hydrogel synthesis shown in Figure 2a). Upon exposure to ultraviolet light, the polymers formed crosslinks between the methacrylate groups, producing a synthetic ECM. These hydrogels (encapsulated VICs, culture media, hydrogel pre-polymers, and LAP) were 3D-bioprinted into sacrificial Pluronic molds using a heated-extruder to produce disc-shaped hydrogels (8.6 mm in diameter × 1.0 mm in height) suitably sized for culture in 12-well plates (Figure 2b,d). Two distinct bioprinting regimes were performed: single-layer hydrogels were printed as above (Figure 2b), while novel dual-layer constructs were produced by serial printing and UV crosslinking of layers (Figure 2c).

### 3.3. Leaflet Layer Mechanical Properties Were Recapitulated in Hydrogels

**Figure 3:**
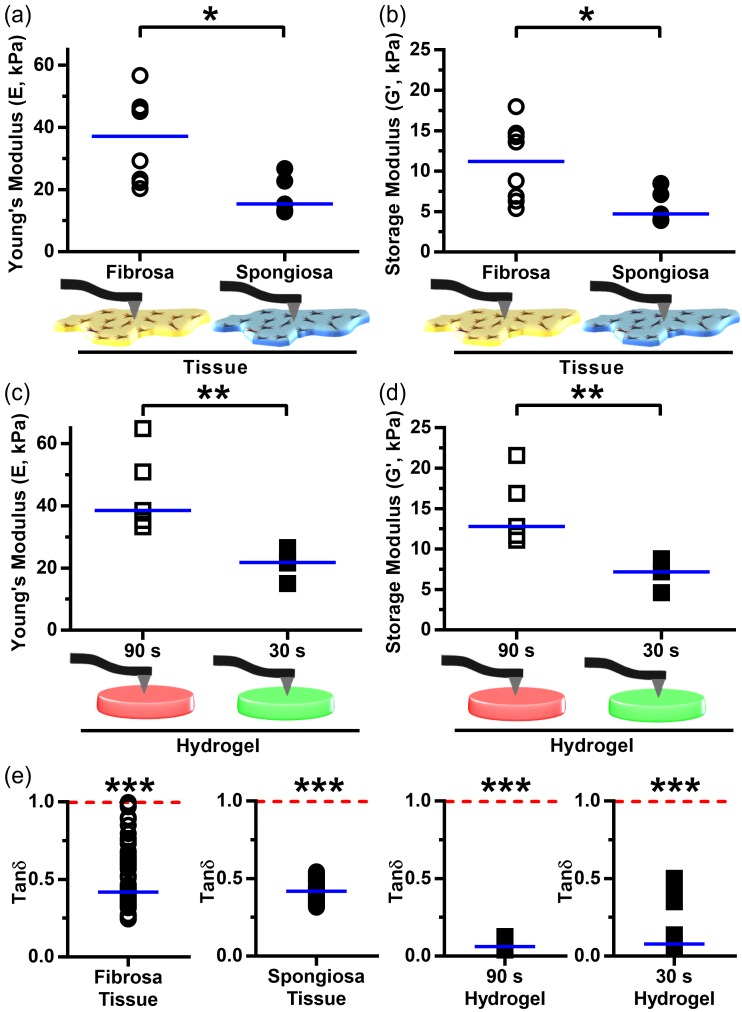
Measurement of layer-specific leaflet mechanical properties and matching of biomechanics with GelMA/HAMA hydrogels: (**a**,**b**) Nanoindentation of the CAVD leaflet layers determined that the median Young’s modulus of the fibrosa layer (37.1 kPa) was significantly higher than that of the spongiosa layer (15.4 kPa), and that the median storage modulus (G′) of the fibrosa layer (11.2 kPa) was significantly higher than that of the spongiosa layer (4.7 kPa); (**c**,**d**) Nanoindentation of hybrid hydrogels found that the median Young’s modulus of the 5% GelMA/1% HAMA hydrogel after UV crosslinking for 90 s (38.5 kPa) was significantly higher than that of the 5% GelMA/1% HAMA/30 s crosslinked hydrogel (21.7 kPa). GelMA/HAMA gels crosslinked for 90 s and 30 s recapitulated Young’s moduli of the fibrosa and spongiosa layers, respectively. G′ of the 90 s crosslinked hydrogel (12.8 kPa) was significantly higher than that of the 30 s crosslinked hydrogel (7.2 kPa). Hydrogel storage moduli again mimicked those properties of the native tissue; (**e**) The tan δ (G′′/G′, loss tangent: viscous vs. elastic deformation) of individual samples within the fibrosa layer, spongiosa layer, 90 s hydrogel, and 30 s hydrogel were all significantly smaller than 1. In comparison to the leaflet layers, tan δ remained lower for both hydrogels. Median shown; * *p* < 0.05, ** *p* < 0.01, *** *p* < 0.001; *n* = 5–9 samples per condition (nine measurements per sample).

Nanoindentation revealed significant differences in mechanical properties between human AV leaflet layers. The fibrosa layer had a significantly higher median Young’s modulus (37.1 kPa) than the spongiosa layer (15.4 kPa) (Figure 3a), while that of the ventricularis was 26.9 kPa (Table S1). The storage modulus G′ had a similar distribution across leaflet layers as that of the Young’s modulus (Figure 3b), and nanoindentation measurements exhibited low measurement-to-measurement variability per tissue sample (Supplemental Figure S1a). Although compressive Young’s moduli were heterogeneous (the fibrosa layer ranged from 20.3–56.7 kPa, spongiosa = 12.8–26.8 kPa, ventricularis = 16.6–33.5 kPa [Table S1]), there were distinct ranges of stiff fibrosa (above ~30 kPa) and soft spongiosa (below ~20 kPa) that were unique to their respective layer. The median Young’s modulus of the intact leaflet (26.7 kPa) lay between the upper and lower bounds of stiff fibrosa and soft spongiosa and highlighted the importance of assessing layer-specific biomechanics when studying layer-specific VIC phenotypes. To assess the relative contributions of viscous and elastic responses in CAVD tissue, quantification of the loss tangent tan δ (loss modulus G′′ over storage modulus G′; Figure 3e) found that tan δ was significantly less than 1 in all layer samples, indicating that elastic deformation was the primary contributor to the complex modulus G* and thus to the Young’s modulus of valvular tissue.

Once compressive moduli of the AV leaflet layers had been determined, we then 3D-bioprinted a series of acellular hybrid hydrogels composed of 5–10% GelMA and 1% HAMA and crosslinked for 30–90 s. Nanoindentation was performed on all hydrogels (Table S2). We found that the 5% GelMA/1% HAMA hydrogel exposed for 30 s had the lowest median modulus (21.7 kPa) and was the best match to the Young’s modulus of the spongiosa layer (Figure 3c). Two hydrogel formulations had Young’s moduli comparable to that of the fibrosa layer: 5% GelMA/1% HAMA/90 s cross-linking (38.5 kPa; Figure 3c), and 6.67% GelMA/1% HAMA/30 s cross-linking (38.6 kPa; Table S2). The former had lower gel-to-gel variability, less overlap in stiffness with the softer 5% GelMA/1% HAMA/30 s hydrogel, and an identical hydrogel composition to that of the 5% GelMA/1% HAMA/30 s hydrogel. As was the case with the AV tissue, hydrogel storage moduli (G′) significantly differed between the 30 s and 90 s 5% GelMA/1% HAMA hydrogels and closely matched those values of their tissue counterparts (Figure 3d). In contrast, tan δ values remained significantly less than one in the hydrogel system (Figure 3e). Unconfined compression testing of 30 s and 90 s 5% GelMA/1% HAMA hydrogels (Supplemental Figure S1b–d) demonstrated that the modulus of 90 s gels was twice that of 30 s gels (as was the case for testing by nanoindentation, Figure 3c). Together, these findings indicated that the specific hydrogel biomechanics closely mimicked those of the individual layers of native AV tissue. On the basis of these data, the 5% GelMA/1% HAMA/30 s hydrogel was utilized as a spongiosa-like (S-like) model and the 5% GelMA/1% HAMA/90 s hydrogel became the fibrosa-like (F-like) model for the remainder of this work.

### 3.4. VIC Encapsulation Affects Hydrogel Mechanics

**Figure 4:**
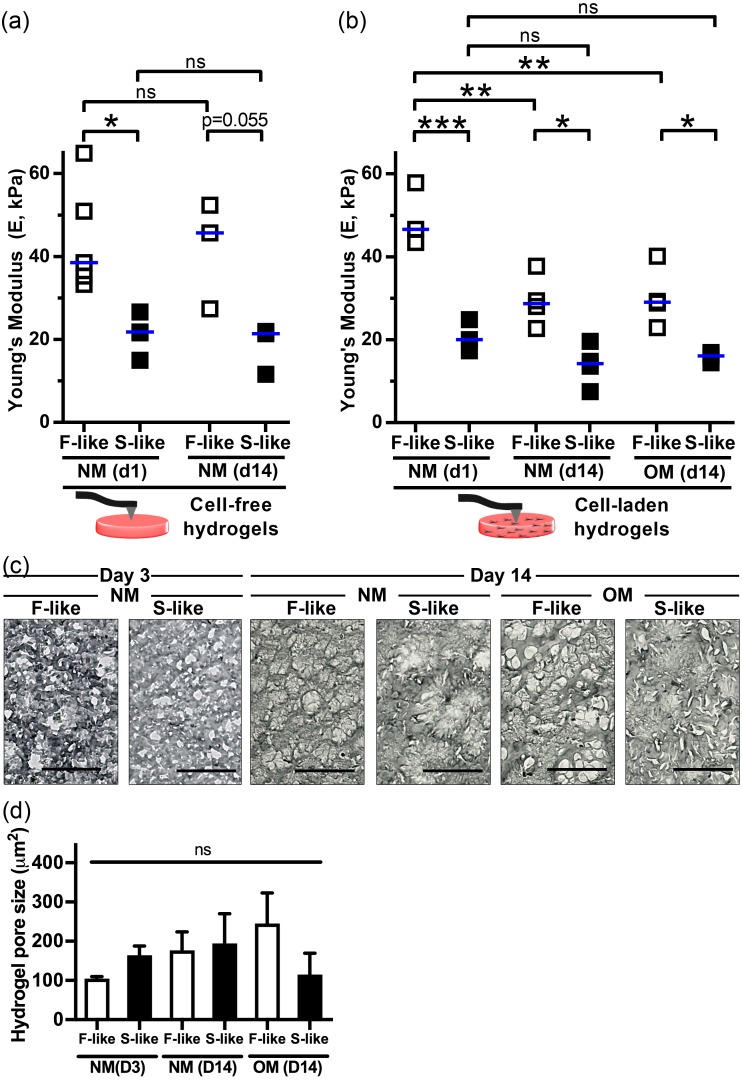
Encapsulation and culture of VICs within fibrosa-like (F-like) but not spongiosa-like (S-like) hydrogels affected hydrogel mechanics: (**a**) The median Young’s modulus of cell-free 3D-bioprinted F-like (5% GelMA/1% HAMA/90 s crosslinking) and S-like (5% GelMA/1% HAMA/30 s crosslinking) hydrogels did not change over 14 days of culture in NM; (**b**) The median Young’s modulus of VIC-laden F-like hydrogels (and not S-like hydrogels) decreased significantly over time in culture (14 days), regardless of NM or OM treatment conditions. Median shown; *n* = 3–5 samples per condition (9 measurements per sample); (**c**,**d**) Representative images and quantification of bright-field microscopy demonstrated that after 3 and 14 days in culture (NM and/or OM), pore sizes of F-like and S-like hydrogels were not significantly different. Mean + SEM; * *p* < 0.05, ** *p* < 0.01; *** *p* < 0.001; *n* = 3 samples per condition; scale bar = 100 µm.

After characterizing acellular hydrogel mechanics, we bioprinted VIC-free and VIC-laden F-like and S-like hydrogels, then cultured them in normal media (NM) or osteogenic media (OM) to assess the long-term impact of VIC encapsulation on hydrogel mechanics. After 14 days in NM (Figure 4a), the median Young’s modulus of VIC-free F-like and S-like hydrogels was unchanged and that of the F-like gel remained significantly higher than that of the S-like gel. At day 1 there was no significant difference between VIC-free and VIC-laden moduli (ANOVA, data not shown). In S-like gels, encapsulation of VICs and culture in NM or OM for 14 days did not affect hydrogel mechanical properties. In contrast to the acellular condition and to VIC-laden S-like hydrogels, VIC-laden F-like gels underwent cell-dependent remodeling and significant reductions in stiffness by the 14-day time point (Figure 4b). Notably however, these cultured VIC-laden F-like hydrogels remained significantly stiffer than their S-like counterparts. By bright-field microscopy (Figure 4c,d), we confirmed that there were no significant changes in VIC-laden hydrogel pore sizes between NM/OM and F-like/S-like conditions after 3 or 14 days of culture, and pore size was consistent with those of 5% GelMA/1% HAMA/30 s crosslinked gels described previously by SEM [19]. Swelling ratio testing of acellular F-like and S-like hydrogels (Supplemental Figure S2) found a significantly increased swelling of S-like hydrogels, likely a result of reduced free volume with greater crosslinking [40].

### 3.5. Preferential Formation of Microcalcification in Fibrosa-Like Hydrogels

**Figure 5:**
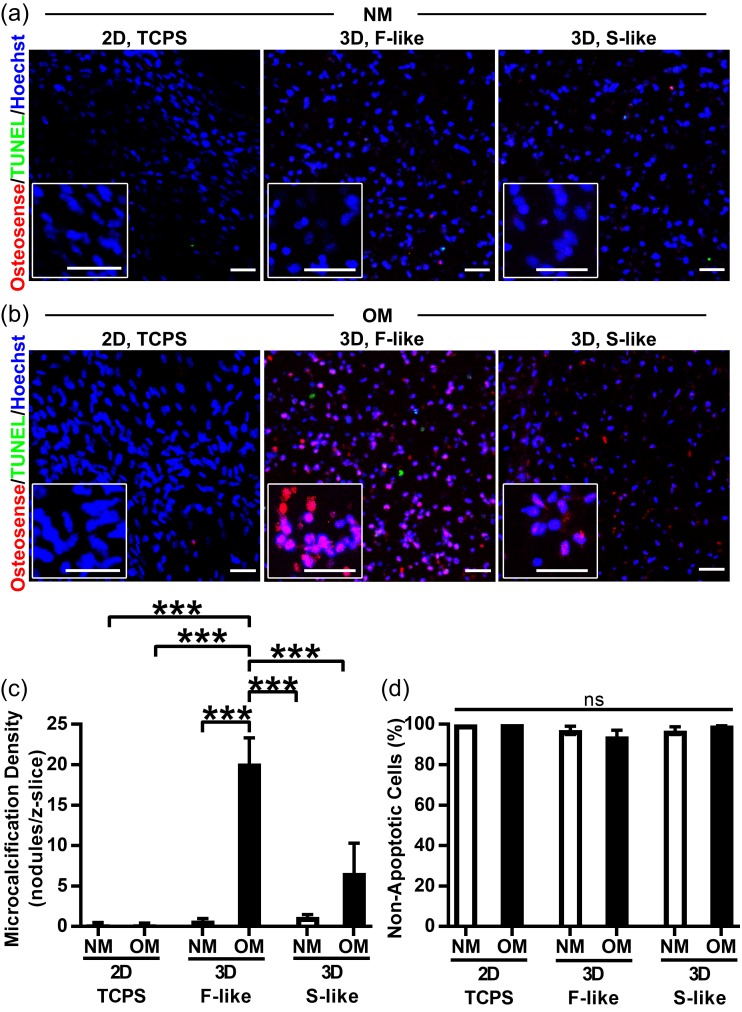
Fibrosa-like hydrogels preferentially induced production of osteogenic microcalcification by VICs. Red = NIRF Osteosense 680 imaging agent (nano- and microcalcifications), green = TUNEL apoptosis assay, blue = Hoechst nuclear stain: (**a**) VICs isolated from non-diseased human AV did not undergo calcification when cultured in NM on/in either 2D tissue culture polystyrene (TCPS), 3D fibrosa-like (F-like) hydrogels, or 3D spongiosa-like (S-like) hydrogels (representative images); (**b**,**c**) Importantly, under OM treatment this naïve VIC population developed significant production of microcalcification only in 3D F-like hydrogels that mimic the biomechanics of the disease-prone layer of the native tissue. OM treatment did not induce significant microcalcification on 2D TCPS or in 3D S-like hydrogels. (**d**) Apoptosis was negligible and unchanged across all conditions, confirming that apoptotic cell death was not the source of preferential calcification in OM-treated F-like hydrogels. Mean + SEM; *** *p* < 0.001; *n* = 3 samples per condition (3 images per sample); scale bar = 50 μm.

After we confirmed our ability to print hydrogels that mimicked the mechanical properties of individual native leaflet layers with encapsulated VICs that maintained an ability to remodel hydrogels, we proceeded to assess the calcifying potential of our 3D-bioprinted model. Naïve VICs derived from non-diseased AV were seeded in 2D well plates or encapsulated in F-like or S-like hydrogels. Under NM treatment (Figure 5a,c), there was no formation of nano- or microcalcification in any culture condition, as assessed by confocal imaging of the calcium binding NIRF imaging agent [41,42]. In contrast, although there was no microcalcification in OM-treated 2D culture and limited amounts in 3D S-like hydrogels, VICs in 3D F-like hydrogels (that recapitulated the biomechanics of the disease-prone fibrosa layer) responded to osteogenic stimuli by significantly increasing microcalcification nodule density vs. all other culture conditions after 14 days (Figure 5b,c). Importantly, apoptotic cell death was (i) almost completely absent and (ii) unchanged across all conditions, thus confirming that apoptotic processes were not driving (or caused by) preferential calcification of OM-treated F-like gels (Figure 5d). We also found minimal apoptosis by day three in culture, shortly after bioprinting/crosslinking (Supplemental Figure S3). Apoptosis levels were again unchanged across all conditions, consistent with an absence of short-term LAP/UV-induced cytotoxicity.

### 3.6. VIC Remodeling of Fibrosa-Like Hydrogels

**Figure 6:**
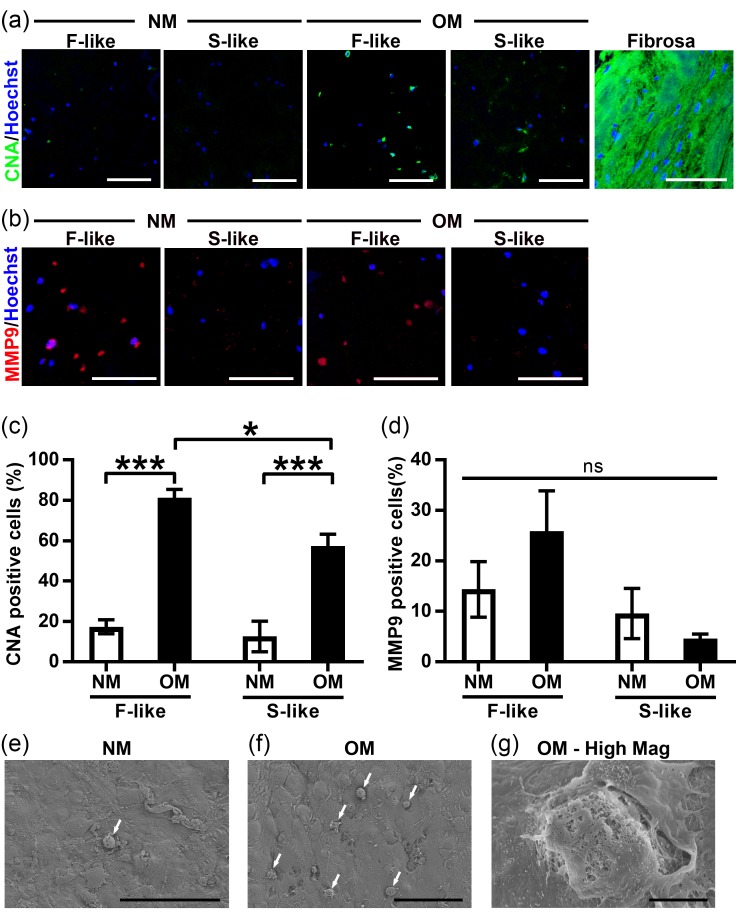
OM stimulation induced VIC pathogenesis in 3D-bioprinted hydrogels: (**a**,**c**) VICs isolated from non-diseased human AV and exposed to OM (and not NM) for 14 days stimulated production of collagen, as shown by representative images of collagen-binding probe (CNA35) fluorescence; (**b**,**d**) OM-treated F-like VICs trended towards increased expression of the ECM-degrading collagenase enzyme MMP-9 (representative immunofluorescence images). Mean ± SEM; * *p* < 0.05, *** *p* < 0.001; *n* = three samples per condition (three images per sample); scale bar = 200 µm; (**e**–**g**) SEM of hydrogels showed that OM treatment drove formation of in vivo-like microcalcific nodules (white arrows), scale bar = 100 µm (**e**,**f**) or 10 µm (**g**).

To further study pathological differentiation and remodeling in our hydrogel model, we treated VIC-laden F-like and S-like hydrogels with NM and OM, then assessed collagen production using the collagen-binding CNA probe [38]. Diseased CAVD fibrosa tissue samples were demonstrably rich in collagen, and OM treatment induced significant increases in collagen production by VICs in both F-like and S-like hydrogels (OM F-like collagen levels were significantly elevated beyond those of OM S-like constructs; Figure 6a,c), with a further substantial increase in collagen production after 28 days in culture (Supplemental Figure S5). As a result of the reduction in F-like stiffness over culture time identified in Figure 4b, we also examined expression of MMP-9, a collagenase/gelatinase previously shown to be upregulated in CAVD [43]. MMP-9 levels trended towards elevation in F-like OM-treated hydrogels (Figure 6b,d). In addition, scanning-electron microscopy (SEM) of VIC-laden hydrogels confirmed that OM treatment drove elevated formation of in vivo-like microcalcific nodules (Figure 6e–g) after 21 days in culture, consistent with the calcium binding NIRF imaging agent staining presented in Figure 5. At day 3, encapsulated VICs were seeded consistently across the height of hydrogels, and this uniform distribution was maintained by day 14 (Supplemental Figure S4).

### 3.7. Integration of Single-Layer Fibrosa-Like and Spongiosa-Like Hydrogels into a 3D-Bioprinted Dual-Layer Construct

**Figure 7:**
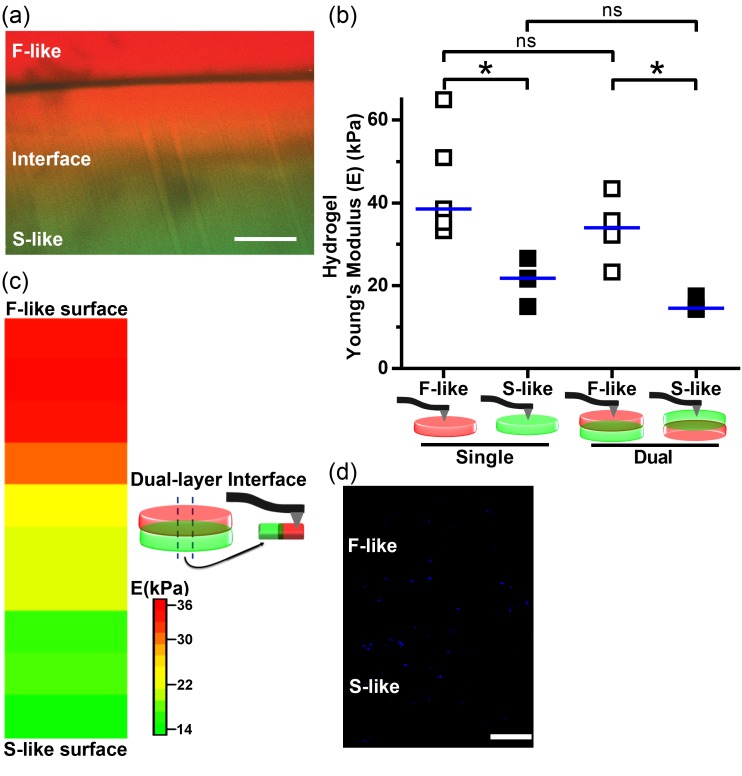
Integration of fibrosa-like (F-like) and spongiosa-like (S-like) single-layer hydrogels into a 3D-bioprinted dual-layer construct maintains leaflet layer-specific mechanical properties: (**a**) Cross-section of a dual-layered 5% GelMA/1% HAMA hydrogel stained with fluorescein-labeled Dextran (shown in red) for the 90 s crosslinked F-like layer, and Texas Red-labeled Dextran (shown in green) for the 30 s crosslinked S-like layer demonstrated layer fusion with a gradient (yellow) at the interface. Scale bar = 200 µm; (**b**) Nanoindentation found no significant differences in the Young’s moduli of the F-like nor S-like layers between single-layer and dual-layer hydrogels; however, significant differences in stiffness between F-like and S-like layers were maintained within dual-layer hydrogel constructs. Median shown; * *p* < 0.05; *n* = 3–4 samples per condition (nine measurements per sample); (**c**) Heatmap of Young’s moduli measured by nanoindentation along the *z*-axis cross section of dual-layer hydrogels demonstrated a stiffness gradient across hydrogel layers; (**d**) Representative cross-sectional image of a Hoescht-stained multi-layered hydrogel with encapsulated VICs at day 14, demonstrating uniform cell encapsulation after long-term culture of dual-layered constructs, scale bar = 200 µm. Median shown; *n* = 3 samples (10 *z*-axis measurements per sample).

Having established 3D-bioprinting of single-layer hydrogels that, individually, modeled the behavior of the fibrosa and spongiosa in vitro, we focused on combining these gels into an integrated model of valvular tissue. Using a strategy of serial bioprinting and partial UV crosslinking, dual-layer constructs that mimicked both the fibrosa and spongiosa were printed as per Figure 2c. When red and green fluorescent Dextran beads were incorporated into individual layers, confocal imaging found evidence of layer fusion with a yellow gradient at the interface (Figure 7a), suggestive of layer intercalation that was also present in the native tissue (see transitions between layers in native AV by Movat’s pentachrome in Figure 1a). The layer interface appeared strong and withstood all processing, handling, and culture without evidence of delamination or degradation. By nanoindentation, we found that F-like or S-like layer Young’s moduli did not differ significantly between their respective single- and dual-layer conformations. Importantly, significant differences between the F-like and S-like layers were maintained when these hydrogel formulations were printed and crosslinked together in dual-layer constructs (Figure 7b). Finally, we used nanoindentation to examine how Young’s modulus varied along the cross-section (*z*-axis) of dual-layer hydrogels (Figure 7c). Three distinct stiffness regimes were identified: a layer with F-like stiffness, followed by a region of intermediate stiffness in the center of the construct, and lastly, an S-like layer. VICs were successfully encapsulated, bioprinted, and cultured for 14 days in these dual-layered hydrogels (Figure 7d). Together, these findings indicated that our 3D-bioprinted multi-layered AV models were potentially suitable for evaluating layer-specific differences in VIC function.

## 4. Discussion

The AV is a complex and dynamic microenvironment (reviewed in C. Y. Yip et al. [44]) with a mechanosensitive resident cell population that actively contributes to disease progression under conditions of altered biomechanics (e.g., hypertension [45] or bicuspid aortic valve [46]). This inherent complexity has retarded efforts to appropriately model CAVD in vitro and negatively impacted drug development for this deadly disease. Here, we successfully paired layer-specific measurements of human AV mechanical properties with 3D-bioprinted hydrogel model systems for the first time.

The tensile properties of the human AV (relevant to cyclic stretch) have been the focus of many studies and have even been performed in a layer-specific manner [12,13,14]. However, tensile factors may be less relevant than valvular substrate stiffness/compressive Young’s moduli, particularly as they relate to static culture models of disease. In non-diseased porcine AVs, a prior study found the fibrosa and ventricularis stiffnesses by micropipette aspiration to be ~22 and 10 kPa, respectively [10], while atomic force microscopy on thin cryosections [47] of porcine valves found the fibrosa, spongiosa, and ventricularis to be ~12, ~4, and ~8 kPa, respectively [15]. Apart from the current study, to our knowledge, the latter is the only other report of spongiosa stiffness in any species. The layer-specific stiffnesses we report here are the first such assessments for the human AV. Notably, we measured stiffnesses of 37.1, 15.4, and 26.9 kPa for the fibrosa, spongiosa, and ventricularis of human AV, respectively, that are higher than those previously reported in animal studies. Two limitations may apply: first, we used surgically excised human AV tissue, whereas prior studies were limited to non-diseased valves from other species. We were careful to (i) omit any regions of macrocalcification and (ii) measured macrocalcifications separately to ensure we could identify and neglect any nanoindentation measurements that inadvertently encompassed calcified areas (Table S1). Along with calcification, CAVD is accompanied by fibrotic changes to all leaflet layers [6], with excess production of disorganized and fragmented collagens that are likely responsible for stiffness increases in diseased vs. non-diseased human AVs. Alteration of valvular stiffness in these diseased yet non-calcified regions of the AV is believed to strongly contribute to biomechanicallymediated pathogenesis (reviewed in J.H. Chen et al. [48]). We omitted any spongiosa samples with notable collagen accumulation but cannot discount that our nanoscale measurements included regions with low-level collagenous changes. Second, although the porcine AV has many similarities to that of the human (including a tri-layered structure) and is often used as a model of AV disease [49], there are substantial differences in ECM composition and disease progression between these two species, as the porcine AV does not calcify readily [50,51]. Together, this indicates that species-specific differences in non-diseased AV layer stiffnesses are likely to exist. Non-diseased human AV tissue is exceedingly difficult to obtain because early/non-diseased human tissue is rarely targeted for surgical replacement; further studies will be needed to delineate the biomechanical properties of non-diseased human valve layers. A prior study found whole-leaflet Young’s moduli of cryopreserved, non-diseased human AVs from older donors to be ~15–20 kPa [52], which is reasonably consistent with the median Young’s modulus of 26.7 kPa we measured in intact CAVD leaflets. We also determined that the loss tangent (tan δ) was significantly skewed towards elastic, and not viscous, deformation in all layers of the human AV.

Gel systems have been used to examine the impact of substrate stiffness modulation on VIC phenotypes. Substrate stiffnesses between 3–144 kPa on 2D polyacrylamide gels modulate responses in Wnt and β-catenin signaling pathways and myofibrogenesis in VICs [10]; however, VICs preferentially undergo calcification and osteogenic differentiation on thick 27 kPa in comparison with thin 113 kPa 2D collagen gels [9]. Others have shown that PEGdma-PLA gels with stiffness of ~120 kPa potentiate myofibrogenic responses in VICs [15]. As with many other cell types [53], VIC mechanobiological responses appear to be regulated by the dimensionality of their surroundings. Microarray studies have identified distinct differences in VIC transcriptional profiles between 2D vs. 3D cultures, particularly in genes associated with cellular structure, polarity, and motility [54]. In 3D, VICs subjected to a transient gradient of photoinitiated thiol-ene polymerization within PEG hydrogels that increased gel moduli transitioned from an initial myofibroblastic nature at 0.24 kPa towards that of a quiescent fibroblast at 13 kPa [18]. Hybrid GelMA/HAMA hydrogels with compressive moduli in the range of 1–3 kPa induced differential synthesis of ECM components and myofibrogenic/osteogenic markers by VICs under media stimulation [11]. However, with few exceptions [10,15], these studies did not specifically match the mechanical properties of their hydrogels to those directly measured from human tissue; instead, they performed comparative high/low analyses. Absolute magnitude of stiffness is likely important to modeling disease; specific stiffness thresholds have been identified for phenotypic transitions (e.g., myofibrogenic, osteogenic, chondrogenic) of VICs [10] and other relevant cell types such as mesenchymal stem cells [8].

Here, we used nanoindentation to directly mimic the compressive moduli of our 3D hydrogel system to that of native human AV layers. We found that, under basal conditions, both fibrosa-like and spongiosa-like stiffnesses of our hydrogel compositions maintained VICs as quiescent fibroblasts. Meanwhile, under osteogenic media stimulation, naïve non-diseased human VICs responded to layer-specific stiffnesses by preferentially producing microcalcifications, secreting collagen, and synthesizing MMP-9 within hydrogels that mimicked the biomechanics of the most disease-prone fibrosa layer of the human AV. Consistent with rarely observed development of CAVD within the spongiosa, S-like hydrogels also exhibited negligible increases in calcification and low collagen production. These in vitro phenotypes replicated key pathways involved in the progression of this disease in vivo: nano- and microcalcifications began to accumulate early in the diseased fibrosa [7,41], eventually forming large macro-scale calcific nodules that inhibited leaflet opening/closure. Fibrotic collagen accumulation is also a hallmark of CAVD [55], and sclerotic changes in the valve leaflets are likely to mediate stiffness increases that further drive pro-myofibro/osteogenic differentiation. In addition, matrix metalloproteinases (e.g., MMP-9) upregulated in CAVD were also found to be increased in our 3D model, suggesting a sequential remodeling of fibrotic ECM [43,56]. In total, our GelMA/HAMA scaffolds were bioactive, allowing us to model VIC ECM remodeling.

One key advantage of bioprinting is the spatial control over hydrogel composition and, accordingly, the direction of biomechanics and cellular responses. Our application of this approach offers the benefit of bioprinting multiple mechanically distinct layers while maintaining a constant hydrogel composition, thereby controlling for any associations between GelMA/HAMA pre-polymer concentrations and VIC responses. This method also simplifies printing, as only a single bioprinter extruder is required to print multiple layers with different biomechanics. The tradeoff necessitated by this approach is differential UV exposure between layers, and resultant differences in hydrogel crosslinking, which has been shown to modulate cellular responses in other tissue types [57]. Importantly, the wavelength of UV light (365 nm) and the power output (2.5 mW/cm^2^) we utilized herein produced negligible levels of apoptosis in our encapsulated VICs (<~5% after short or long-term culture) and are well-documented to be compatible with VIC viability and quiescence/myofibrogenic/osteogenic responses, depending upon the type of exogenous stimulation [19,20]. If disparities in UV dosage are a concern for future applications of this approach, we describe here an alternative formulation of 6.67% GelMA/1% HAMA (Table S2) that matches the higher fibrosa stiffness while requiring only 30 s of UV exposure (consistent with the 5% GelMA/1% HAMA/30 s UV we utilized for the S-like layer). The use of sacrificial molds enabled us to have consistent and highly customizable control over hydrogel size (i.e., not limited to the standard well plate sizes), negated any meniscus formation, and allowed for free-floating hydrogels. The latter ensured hydrogels could be easily manipulated for biomechanical testing, culture under complex conditions (e.g., stretch/shear bioreactors), confocal imaging, and histopathological sectioning. Nutrient and oxygen diffusion can also occur via all surfaces of the hydrogel. We also demonstrated that individual hydrogel layers with different mechanical properties could be sequentially 3D-bioprinted into complex multilayered constructs while maintaining their unique individual biomechanical properties. Such integrated hydrogel models hold promise for future studies of layer-layer interactions/signaling; the use of bioprinting described herein promises to enable automated multi-well fabrication of hydrogel arrays suitable for higher experimental throughput, testing under hemodynamic loading, and drug target screening. Beyond their compatibility with such pharmaceutical technologies, this “organ-on-a-chip”-like approach also brings us a step closer to non-animal alternatives for preclinical research in CAVD [58].

## 5. Conclusions

In summary, we demonstrate the bioprinting process of a 3D in vitro model of human CAVD using specific concentrations of GelMA/HAMA hydrogels that enabled us to mimic the ECM of native tissue and maintain VIC quiescence under basal conditions. The present study is the first to quantify compressive mechanical properties of each layer of the human AV and recapitulate those layer-specific biomechanics in vitro. This work also found that induction of nano- and microcalcification and pathological differentiation in naïve, non-diseased VICs can be driven by layer-specific mechanical properties matching those of the disease-prone fibrosa layer of the human AV. Together, these findings establish a novel 3D model for the further study of valvular mechanobiology and are an important step towards high-throughput screening of drug targets for CAVD in a biologically-relevant model of disease. Broadly, we present a generalizable strategy for matching tissue mechanical properties with scalable 3D-bioprinting of in vitro models in tissues with complex ECM composition and mechanosensitive resident cell population(s).

## Supplementary Materials

The following are available online at http://www.mdpi.com/2079-4991/8/5/296/s1.

## References

[1] Messika-Zeitoun D., Bielak L.F., Peyser P.A., Sheedy P.F., Turner S.T., Nkomo V.T., Breen J.F., Maalouf J., Scott C., Tajik A.J. (2007). Aortic valve calcification: Determinants and progression in the population. Arterioscler. Thromb. Vasc. Biol..

[2] Aikawa E., Libby P. (2017). A rock and a hard place: Chiseling away at the multiple mechanisms of aortic stenosis. Circulation.

[3] Hutcheson J.D., Blaser M.C., Aikawa E. (2017). Giving calcification its due: Recognition of a diverse disease: A first attempt to standardize the field. Circ. Res..

[4] Hutcheson J.D., Goettsch C., Bertazzo S., Maldonado N., Ruiz J.L., Goh W., Yabusaki K., Faits T., Bouten C., Franck G. (2016). Genesis and growth of extracellular-vesicle-derived microcalcification in atherosclerotic plaques. Nat. Mater..

[5] Yutzey K.E., Demer L.L., Body S.C., Huggins G.S., Towler D.A., Giachelli C.M., Hofmann-Bowman M.A., Mortlock D.P., Rogers M.B., Sadeghi M.M. (2014). Calcific aortic valve disease: A consensus summary from the alliance of investigators on calcific aortic valve disease. Arterioscler. Thromb. Vasc. Biol..

[6] Otto C.M., Kuusisto J., Reichenbach D.D., Gown A.M., O’Brien K.D. (1994). Characterization of the early lesion of ‘degenerative’ valvular aortic stenosis. Histological and immunohistochemical studies. Circulation.

[7] Yabusaki K., Hutcheson J.D., Vyas P., Bertazzo S., Body S.C., Aikawa M., Aikawa E. (2016). Quantification of calcified particles in human valve tissue reveals asymmetry of calcific aortic valve disease development. Front. Cardiovasc. Med..

[8] Engler A.J., Sen S., Sweeney H.L., Discher D.E. (2006). Matrix elasticity directs stem cell lineage specification. Cell.

[9] Yip C.Y., Chen J.H., Zhao R., Simmons C.A. (2009). Calcification by valve interstitial cells is regulated by the stiffness of the extracellular matrix. Arterioscler. Thromb. Vasc. Biol..

[10] Chen J.H., Chen W.-L., Sider K., Yip C.Y., Simmons C.A. (2011). Beta-catenin mediates mechanically-regulated, tgf-beta1-induced myofibroblast differentiation of aortic valve interstitial cells. Arterioscler. Thromb. Vasc. Biol..

[11] Duan B., Yin Z., Hockaday Kang L., Magin R.L., Butcher J.T. (2016). Active tissue stiffness modulation controls valve interstitial cell phenotype and osteogenic potential in 3d culture. Acta Biomater..

[12] Balguid A., Rubbens M.P., Mol A., Bank R.A., Bogers A.J., van Kats J.P., de Mol B.A., Baaijens F.P., Bouten C.V. (2007). The role of collagen cross-links in biomechanical behavior of human aortic heart valve leaflets--relevance for tissue engineering. Tissue Eng..

[13] Stella J.A., Sacks M.S. (2007). On the biaxial mechanical properties of the layers of the aortic valve leaflet. J. Biomech. Eng..

[14] Vesely I., Noseworthy R. (1992). Micromechanics of the fibrosa and the ventricularis in aortic valve leaflets. J. Biomech..

[15] Hinderer S., Seifert J., Votteler M., Shen N., Rheinlaender J., Schaffer T.E., Schenke-Layland K. (2014). Engineering of a bio-functionalized hybrid off-the-shelf heart valve. Biomaterials.

[16] Blaser M.C., Wei K., Adams R.L., Zhou Y.Q., Caruso L.L., Mirzaei Z., Lam A., Tam R.K., Zhang H., Heximer S.P. (2018). Deficiency of natriuretic peptide receptor 2 promotes bicuspid aortic valves, aortic valve disease, left ventricular dysfunction, and ascending aortic dilatations in mice. Circ. Res..

[17] Schlotter F., Halu A., Goto S., Blaser M.C., Body S.C., Lee L.H., Higashi H., DeLaughter D.M., Hutcheson J.D., Vyas P. (2018). Spatiotemporal multi-omics mapping generates a molecular atlas of the aortic valve and reveals networks driving disease. Circulation.

[18] Mabry K.M., Lawrence R.L., Anseth K.S. (2015). Dynamic stiffening of poly(ethylene glycol)-based hydrogels to direct valvular interstitial cell phenotype in a three-dimensional environment. Biomaterials.

[19] Hjortnaes J., Camci-Unal G., Hutcheson J.D., Jung S.M., Schoen F.J., Kluin J., Aikawa E., Khademhosseini A. (2015). Directing valvular interstitial cell myofibroblast-like differentiation in a hybrid hydrogel platform. Adv. Healthc. Mater..

[20] Hjortnaes J., Goettsch C., Hutcheson J.D., Camci-Unal G., Lax L., Scherer K., Body S., Schoen F.J., Kluin J., Khademhosseini A. (2016). Simulation of early calcific aortic valve disease in a 3d platform: A role for myofibroblast differentiation. J. Mol. Cell. Cardiol..

[21] Murphy S.V., Atala A. (2014). 3d bioprinting of tissues and organs. Nat. Biotechnol..

[22] van der Ven C.F., Wu P.J., Tibbitt M.W., van Mil A., Sluijter J.P., Langer R., Aikawa E. (2017). In vitro 3d model and mirna drug delivery to target calcific aortic valve disease. Clin. Sci..

[23] Xu F., Celli J., Rizvi I., Moon S., Hasan T., Demirci U. (2011). A three-dimensional in vitro ovarian cancer coculture model using a high-throughput cell patterning platform. Biotechnol. J..

[24] Norotte C., Marga F.S., Niklason L.E., Forgacs G. (2009). Scaffold-free vascular tissue engineering using bioprinting. Biomaterials.

[25] Flores R.L., Liss H., Raffaelli S., Humayun A., Khouri K.S., Coelho P.G., Witek L. (2017). The technique for 3d printing patient-specific models for auricular reconstruction. J. Craniomaxillofac. Surg..

[26] Bhora F.Y., Lewis E.E., Rehmani S.S., Ayub A., Raad W., Al-Ayoubi A.M., Lebovics R.S. (2017). Circumferential three-dimensional-printed tracheal grafts: Research model feasibility and early results. Ann. Thorac. Surg..

[27] Levato R., Webb W.R., Otto I.A., Mensinga A., Zhang Y., van Rijen M., van Weeren R., Khan I.M., Malda J. (2017). The bio in the ink: Cartilage regeneration with bioprintable hydrogels and articular cartilage-derived progenitor cells. Acta Biomater..

[28] Duan B., Hockaday L.A., Kang K.H., Butcher J.T. (2013). 3D bioprinting of heterogeneous aortic valve conduits with alginate/gelatin hydrogels. J. Biomed. Mater. Res. Part A.

[29] Qian Z., Wang K., Liu S., Zhou X., Rajagopal V., Meduri C., Kauten J.R., Chang Y.H., Wu C., Zhang C. (2017). Quantitative prediction of paravalvular leak in transcatheter aortic valve replacement based on tissue-mimicking 3d printing. JACC. Cardiovasc. Imaging.

[30] Nichol J.W., Koshy S.T., Bae H., Hwang C.M., Yamanlar S., Khademhosseini A. (2010). Cell-laden microengineered gelatin methacrylate hydrogels. Biomaterials.

[31] Burdick J.A., Chung C., Jia X., Randolph M.A., Langer R. (2005). Controlled degradation and mechanical behavior of photopolymerized hyaluronic acid networks. Biomacromolecules.

[32] Akhtar R., Schwarzer N., Sherratt M.J., Watson R.E., Graham H.K., Trafford A.W., Mummery P.M., Derby B. (2009). Nanoindentation of histological specimens: Mapping the elastic properties of soft tissues. J. Mater. Res..

[33] Akhtar R., Draper E.R., Adams D.J., Pfaff H. (2016). Complex Shear Modulus of Hydrogels Using a Dynamic Nanoindentation Method.

[34] Cohen S.R., Kalfon-Cohen E. (2013). Dynamic nanoindentation by instrumented nanoindentation and force microscopy: A comparative review. Beilstein J. Nanotechnol..

[35] Choi A.P.C., Zheng Y.P. (2005). Estimation of young’s modulus and poisson’s ratio of soft tissue from indentation using two different-sized indentors: Finite element analysis of the finite deformation effect. Med. Biol. Eng. Comput..

[36] Hamid M.S., Sabbah H.N., Stein P.D. (1987). Vibrational analysis of bioprosthetic heart valve leaflets using numerical models: Effects of leaflet stiffening, calcification, and perforation. Circ. Res..

[37] Riem Vis P.W., Bouten C.V., Sluijter J.P., Pasterkamp G., van Herwerden L.A., Kluin J. (2010). Platelet-lysate as an autologous alternative for fetal bovine serum in cardiovascular tissue engineering. Tissue Eng. Part A.

[38] Aper S.J., van Spreeuwel A.C., van Turnhout M.C., van der Linden A.J., Pieters P.A., van der Zon N.L., de la Rambelje S.L., Bouten C.V., Merkx M. (2014). Colorful protein-based fluorescent probes for collagen imaging. PLoS ONE.

[39] Thubrikar M. (1990). The Aortic Valve.

[40] Schmidt M., Rodler N., Miesbauer O., Rojahn M., Vogel T., Dörfler R., Kucukpinar E., Langowski H.-C. (2012). Adhesion and barrier performance of novel barrier adhesives used in multilayered high-barrier laminates. J. Adhes. Sci. Technol..

[41] Aikawa E., Nahrendorf M., Sosnovik D., Lok V.M., Jaffer F.A., Aikawa M., Weissleder R. (2007). Multimodality molecular imaging identifies proteolytic and osteogenic activities in early aortic valve disease. Circulation.

[42] Aikawa E., Nahrendorf M., Figueiredo J.L., Swirski F.K., Shtatland T., Kohler R.H., Jaffer F.A., Aikawa M., Weissleder R. (2007). Osteogenesis associates with inflammation in early-stage atherosclerosis evaluated by molecular imaging in vivo. Circulation.

[43] Satta J., Oiva J., Salo T., Eriksen H., Ohtonen P., Biancari F., Juvonen T.S., Soini Y. (2003). Evidence for an altered balance between matrix metalloproteinase-9 and its inhibitors in calcific aortic stenosis. Ann. Thorac. Surg..

[44] Yip C.Y., Simmons C.A. (2011). The aortic valve microenvironment and its role in calcific aortic valve disease. Cardiovasc. Pathol..

[45] Yap C.H., Kim H.S., Balachandran K., Weiler M., Haj-Ali R., Yoganathan A.P. (2010). Dynamic deformation characteristics of porcine aortic valve leaflet under normal and hypertensive conditions. Am. J. Physiol. Heart Circ. Physiol..

[46] Szeto K., Pastuszko P., del Alamo J.C., Lasheras J., Nigam V. (2013). Bicuspid aortic valves experience increased strain as compared to tricuspid aortic valves. World J. Pediat. Congenit. Heart Surg..

[47] Sewell-Loftin M.K., Brown C.B., Baldwin H.S., Merryman W.D. (2012). A novel technique for quantifying mouse heart valve leaflet stiffness with atomic force microscopy. J. Heart Valve Dis..

[48] Chen J.H., Simmons C.A. (2011). Cell-matrix interactions in the pathobiology of calcific aortic valve disease: Critical roles for matricellular, matricrine, and matrix mechanics cues. Circ. Res..

[49] Sider K.L., Blaser M.C., Simmons C.A. (2011). Animal models of calcific aortic valve disease. Int. J. Inflamm..

[50] Sim E.K., Muskawad S., Lim C.S., Yeo J.H., Lim K.H., Grignani R.T., Durrani A., Lau G., Duran C. (2003). Comparison of human and porcine aortic valves. Clin. Anat..

[51] Sider K.L., Zhu C., Kwong A.V., Mirzaei Z., de Lange C.F., Simmons C.A. (2014). Evaluation of a porcine model of early aortic valve sclerosis. Cardiovasc. Pathol..

[52] van Geemen D., Soares A.L., Oomen P.J., Driessen-Mol A., Janssen-van den Broek M.W., van den Bogaerdt A.J., Bogers A.J., Goumans M.J., Baaijens F.P., Bouten C.V. (2016). Age-dependent changes in geometry, tissue composition and mechanical properties of fetal to adult cryopreserved human heart valves. PLoS ONE.

[53] Riedl A., Schlederer M., Pudelko K., Stadler M., Walter S., Unterleuthner D., Unger C., Kramer N., Hengstschlager M., Kenner L. (2017). Comparison of cancer cells in 2d vs 3d culture reveals differences in akt-mtor-s6k signaling and drug responses. J. Cell Sci..

[54] Mabry K.M., Payne S.Z., Anseth K.S. (2016). Microarray analyses to quantify advantages of 2d and 3d hydrogel culture systems in maintaining the native valvular interstitial cell phenotype. Biomaterials.

[55] Simard L., Cote N., Dagenais F., Mathieu P., Couture C., Trahan S., Bosse Y., Mohammadi S., Page S., Joubert P. (2017). Sex-related discordance between aortic valve calcification and hemodynamic severity of aortic stenosis: Is valvular fibrosis the explanation?. Circ. Res..

[56] Balachandran K., Sucosky P., Jo H., Yoganathan A.P. (2009). Elevated cyclic stretch alters matrix remodeling in aortic valve cusps: Implications for degenerative aortic valve disease. Am. J. Physiol. Heart Circ. Physiol..

[57] Bryant S.J., Chowdhury T.T., Lee D.A., Bader D.L., Anseth K.S. (2004). Crosslinking density influences chondrocyte metabolism in dynamically loaded photocrosslinked poly(ethylene glycol) hydrogels. Ann. Biomed. Eng..

[58] Otto I.A., Breugem C.C., Malda J., Bredenoord A.L. (2016). Ethical considerations in the translation of regenerative biofabrication technologies into clinic and society. Biofabrication.

