# Supplementary material for: Engineering a 3D-Bioprinted Model of Human Heart Valve Disease Using Nanoindentation-Based Biomechanics
Source: Nanomaterials (Basel). 2018 May 3;8(5):296. doi: 10.3390/nano8050296 (PMC5977310; doi:10.3390/nano8050296)
Supplement: Supplementary file 1 [file nanomaterials-08-00296-s001.pdf]

Supplementary Materials

Table S1. Compressive Young's modulus (E), Storage modulus (G'), Loss modulus (G''), Complex modulus (G*), and tan δ of all valve layer samples.

Layer	E (kPa)		G' (kPa)		G'' (kPa)		G* (kPa)		tan δ	
	Median	Range	Median	Range	Median	Range	Median	Range	Median	Range
Whole Valve	26.7	22.2-43.2	8.2	7.1-12.6	3.0	2.2-6.7	8.9	7.8-14.8	0.35	0.29-0.48
Fibrosa	37.1	20.3 - 56.7	11.2	5.4-18.0	4.4	2.8-6.3	12.4	6.8-18.9	0.42	0.25-0.75
Spongiosa	15.4	12.8-26.8	4.7	3.9-8.5	2.0	1.9-2.9	5.1	4.3-8.9	0.43	0.33-0.49
Ventricularis	26.9	16.6-33.5	8.7	5.4-13.2	3.4	2.0-4.0	9.0	1.9-11.2	0.39	0.38-0.73
Calcification	670.1	259.5-1080.7	212	78.6-345.3	69.2	36.2-102.1	223.4	86.5-360.2	0.32	0.29-0.36

Table S2. Compressive Young's modulus (E), Storage modulus (G'), Loss modulus (G''), Complex modulus (G*), and tan δ of samples with different hydrogel formulations.

Cross-linking time (s)	GelMA % [1]	E (kPa)		G' (kPa)		G'' (kPa)		G* (kPa)		tan δ	
		Median	Range	Median	Range	Median	Range	Median	Range	Median	Range
30	5	21.8	15.0 - 26.6	7.2	4.6 - 8.8	0.6	0.4 - 1.9	7.3	5.0 - 8.9	0.08	0.06-0.42
30	6.67	38.6	23.6 - 60.1	10.5	7.8 - 20.0	0.9	0.4 - 1.9	10.7	7.9 - 20.0	0.09	0.04-0.15
30	8.33	50.2	38.2 - 54.1	16.7	12.7 - 18.0	1.3	0.6 - 2.0	16.7	12.7 - 18.0	0.08	0.05-0.11
30	10	49.1	28.3 - 76.4	15.9	9.4 - 25.5	1.7	0.8 - 4.0	16.4	9.4 - 25.5	0.13	0.09-0.24
90	5	38.5	33.3 - 64.9	12.8	11.1 - 21.6	0.9	0.8 - 1.0	12.8	11.1 - 21.6	0.06	0.05-0.09
90	6.67	50.4	29.3 - 70.2	16.8	8.9 - 23.3	2.3	1.3 - 4.4	16.8	9.8 - 23.4	0.12	0.08-0.49
90	8.33	53	42.2 - 63.1	17.2	13.0 - 19.4	3.6	1.8 - 9.7	17.7	14.1 - 21.0	0.26	0.08-0.50
90	10	53.7	34.8 - 77.8	16.8	9.2 - 25.9	3.5	1.6 - 7.1	17.9	11.6 - 25.9	0.26	0.06-0.78

[1]All hydrogels contain 1% HAMA in addition to the stated % of GelMA.

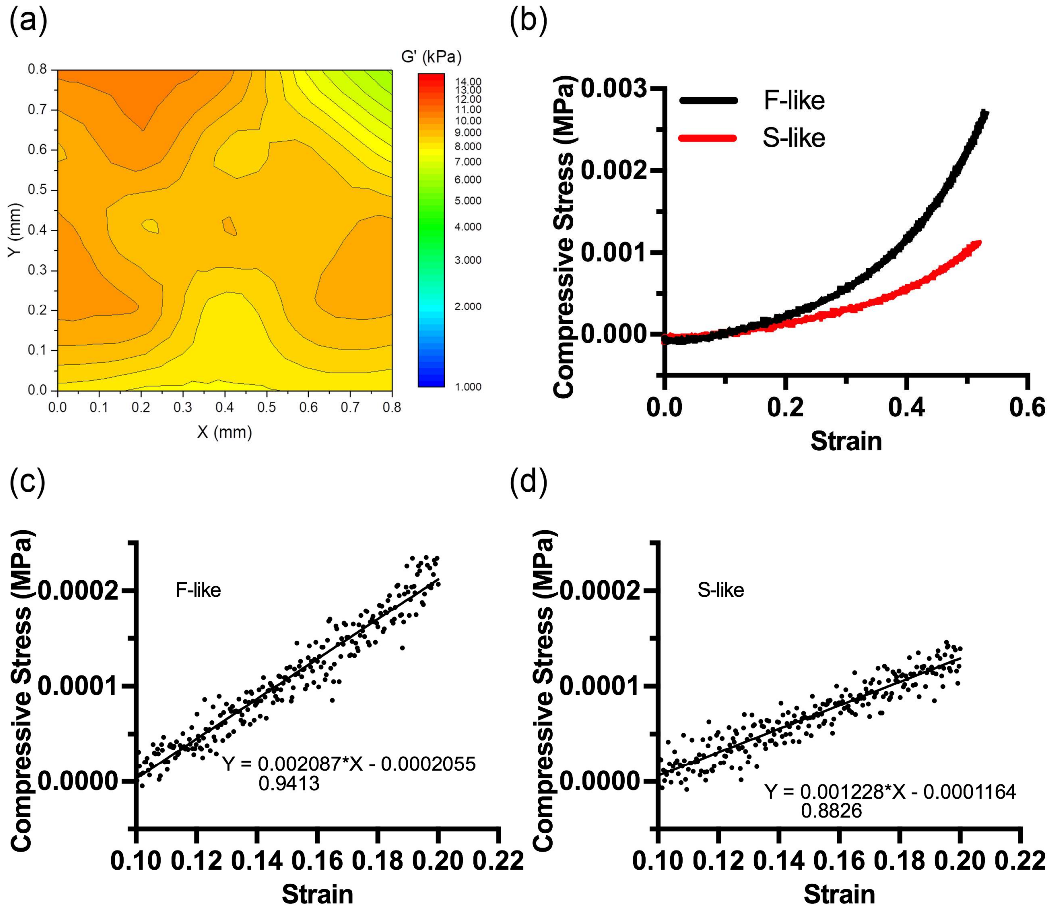

Figure S1. Mechanical testing of aortic valve tissue and GelMA/HAMA hydrogels demonstrated the validity of bulk nanoindentation measurements: (**a**) Heat map of storage moduli (G′) generated from 9 nanoindentations performed across the surface of a valve leaflet layer demonstrated uniformity of nanoindentation-measured G′ values, (**b**–**d**) Stress/strain curves generated by unconfined compression testing of F-like and S-like hydrogels showed that the modulus of F-like hydrogels was ~2x that of S-like hydrogels, consistent with moduli measured by nanoindentation. Parts **c** and **d** are magnification of the linear region of the loading curves in part **b.** Calculation of moduli by linear regression in these regions found a ~2x increase in loading curve slope between F-like and S-like hydrogels.

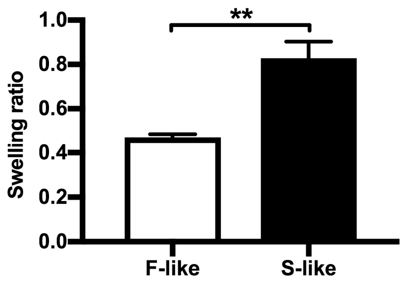

Figure S2. 24-hour hydrogel swelling ratios: There was a significant increase in the swelling ratio of acellular S-like hydrogels vs. those of F-like hydrogels after 24 hours in PBS at room temperature. Swelling ratio = ($weight_{24hr}$ – $weight_{0hr}$)/$weight_{24hr}$; n = 4 samples per condition, ** $p < 0.01$.

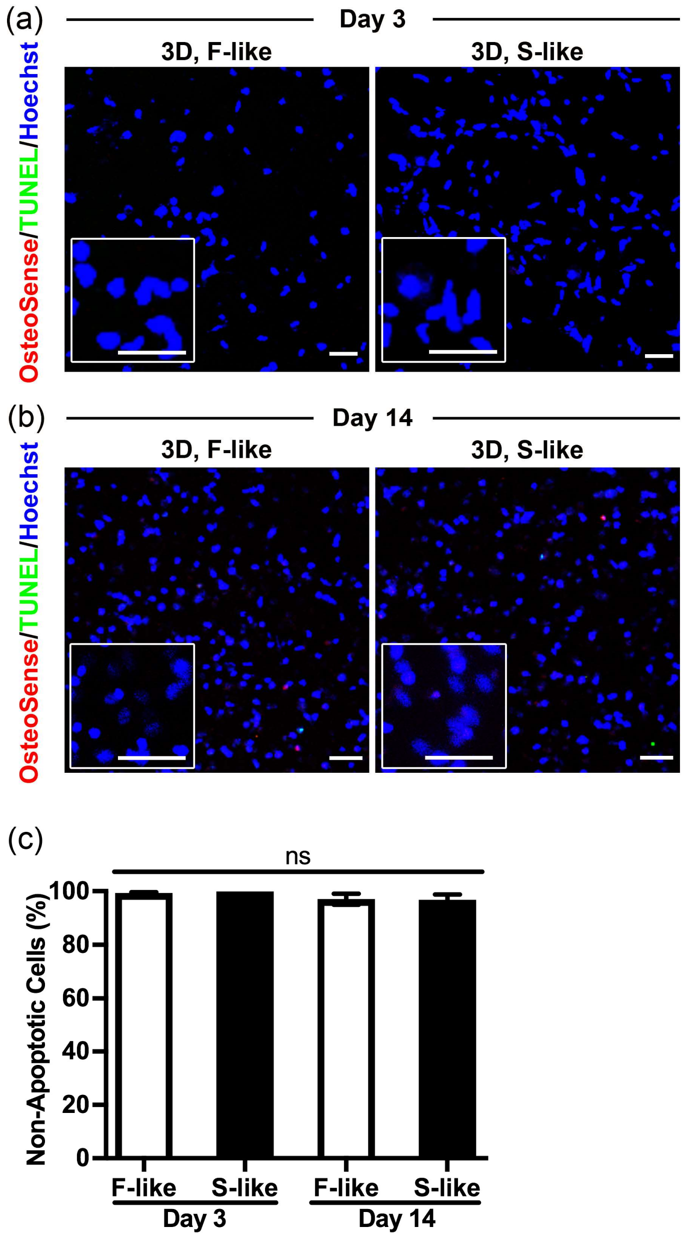

Figure S3. Low levels of short- or long-term apoptotic cell death in 3D-bioprinted hydrogels, green = TUNEL apoptosis assay, blue = Hoechst nuclear stain: (**a**–**c**) VICs isolated from non-diseased human AV and cultured in NM showed negligible levels of apoptosis at 3 (**a**) or 14 (**b**) days after bioprinting. There were no significant differences in apoptosis between F-like or S-like hydrogels, nor between the day 3 and day 14 time points. n = 3 samples per condition (3 images per sample); scale bar = 50 µm. **Note:** Part **b** and associated quantification data is duplicated from Figure 5a, to enable direct comparison here.

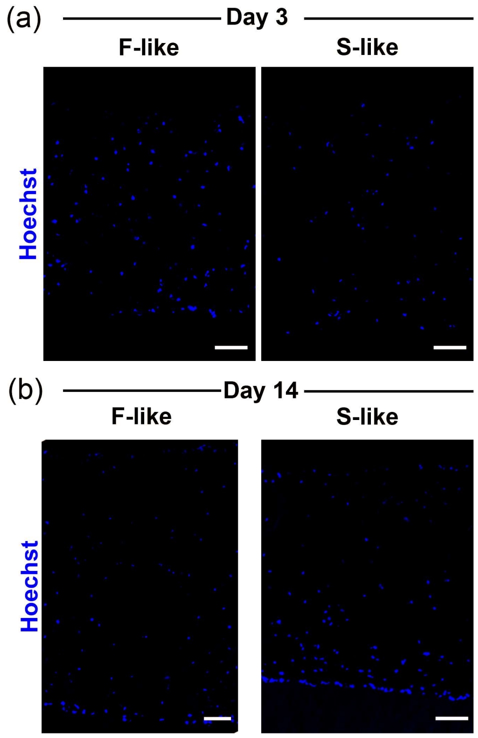

Figure S4. Uniformity of encapsulated VIC distributions in 3D-bioprinted hydrogels, blue = Hoechst nuclear stain: (**a**/**b**) Representative cross-sectional images of cell distribution in F-like and S-like hydrogels after 3 (**a**) and 14 (**b**) days in NM culture demonstrated evenly distributed initial VIC seeding was maintained over long-term culture of hydrogels; scale bar = 100 µm.

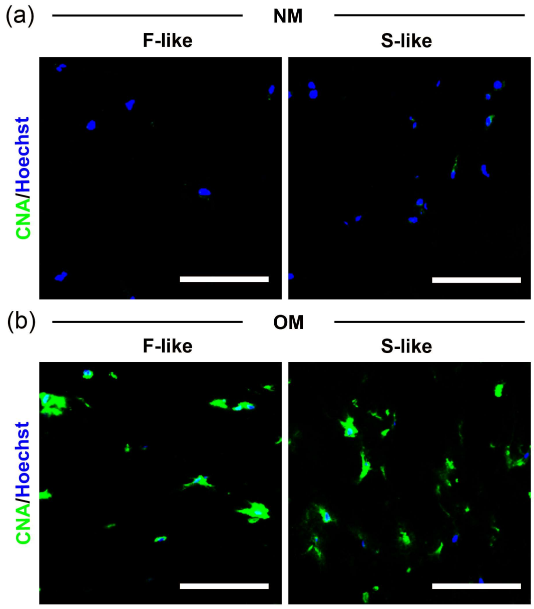

Figure S5. OM stimulation induced marked VIC collagen secretion after 28 days in hydrogel culture: (**a**,**b**) VICs isolated from non-diseased human AV and exposed to OM (and not NM) for 28 days stimulated substantial production of collagen, as shown by representative images of collagen-binding probe (CNA35) fluorescence (green); scale bar = 100 µm.
